# Comprehensive insights into the genetic background of Chinese populations using Y chromosome markers

**DOI:** 10.1098/rsos.230814

**Published:** 2023-09-20

**Authors:** Jienan Li, Feng Song, Min Lang, Mingkun Xie

**Affiliations:** ^1^ Department of Forensic Science, School of Basic Medical Sciences, Central South University, Changsha, Hunan Province, People's Republic of China; ^2^ Institute of Forensic Medicine, West China School of Basic Medical Sciences & Forensic Medicine, Sichuan University, Chengdu, People's Republic of China; ^3^ Sichuan University Law School, Sichuan University, Chengdu, People's Republic of China; ^4^ Department of Obstetrics, Xiangya Hospital, Central South University, 87 Xiangya Road, Changsha, Hunan 410008, People's Republic of China

**Keywords:** Y-STR, Y haplogroups, paternal lineages, Chinese, genetic markers

## Abstract

China is located in East Asia. With a high genetic and cultural diversity, human migration in China has always been a hot topic of genetics research. To explore the origins and migration routes of Chinese males, 3333 Chinese individuals (Han, Hui, Mongolia, Yi and Kyrgyz) with 27 Y-STRs and 143 Y-SNPs from published literature were analysed. Our data showed that there are five dominant haplogroups (O2-M122, O1-F265, C-M130, N-M231, R-M207) in China. Combining analysis of haplogroup frequencies, geographical positions and time with the most recent common ancestor (TMRCA), we found that haplogroups C-M130, N-M231 and R1-M173 and O1a-M175 probably migrated into China via the northern route. Interestingly, we found that haplogroup C*-M130 in China may originate in South Asia, whereas the major subbranches C2a-L1373 and C2b-F1067 migrated from northern China. The results of BATWING showed that the common ancestry of Y haplogroup in China can be traced back to 17 000 years ago, which was concurrent with global temperature increases after the Last Glacial Maximum.

## 1. Introduction

Covering a land area of about 9.6 million square kilometres, China ranks as the third largest country in the world, following Russia and Canada. It is a multi-ethnic country with a long history of civilization. Archaeologists have discovered that Chinese civilization originated 5800 years ago along the Yellow River, the middle and lower reaches of the Yangtze River and the Western Liaohe River [1]. About 18.46% of the world's population lives in China, and more than 72 languages in five linguistic families (Sino-tibetan, Altaic, Austronesian, Austroasiatic and Indo-European languages) are spoken there (http://www.moe.gov.cn/jyb_sjzl/wenzi/). As a consequence, China has long been one of the best places to study human evolution, civilization, and genetics.

The male specific region of the Y chromosome (MSY) with paternal inheritance and lack of recombination is a powerful tool for inferring paternal ancestry and identifying paternal genealogy [2]. As one kind of marker on the Y chromosome, Y chromosome short tandem repeats (Y-STRs) with a high mutation rate (1.0 × 10^−4^ −1.0 × 10^−2^ mutations/generation) can generate unique haplotypes which have been used in sexual assault cases and familial searching [3,4]. In 2022, Nothnagel *et al.* explored genetic variation among geographical regions and among ethnic groups through information on 17 commonly used Y-STRs in the YHRD database [5]. Y-chromosomal single nucleotide polymorphisms (Y-SNPs) with a lower mutation rate can define stable haplogroups and build robust phylogenies [6]. To the best of our knowledge, the Y haplogroup phylogenetic tree was illustrated in 1997 [7]. With the development of high-throughput sequencing technology, more Y-SNPs have been explored [8]. Y-SNPs are highly suitable for paternal bio-geographic ancestry inference and paternal lineage identification [9].

Combining analysis of Y-SNP and STR has been proved to be a good strategy to predict population sub-structure and to explore human origins/migrations [10]. In 2010, Shi *et al*. proposed a model of human expansion of East African origin by studying the demographic history of human males with Y-SNP and STR markers from 51 populations [11]. Several studies have been conducted on Y chromosome genetic markers for specific Chinese ethnic groups. However, the study of genetic structure variation among different populations within China may provide clues for future human migration in China. As different populations have different demographic structures and origins, more comprehensive analysis of demographic data is needed to gain insight into the human evolutionary and migratory history of Chinese people. Meanwhile, these works are important for inferring paternal genealogy in forensics.

In this study, we conducted a detailed analysis of five ethnic groups (Han, Hui, Mongolian, Yi and Kyrgyz), which account for 93.51% of the total population of China. We focused on the following questions: Does geography/ethnicity play an essential role in genetic affinity within China? What is the genetic relationship between Chinese populations and its surrounding populations? Can we find any evidence of early human activity among Chinese ancestors? If so, is it possible to characterize them?

## 2. Material and methods

### 2.1. Samples

We searched PubMed with the keywords ‘Y-STR’, (Y-SNP’ or ‘Y haplogroup), and (Chinese’ or ‘China') from 2019 to 2021. Inclusion criteria were as follows: (1) containing at least the 27 Y-STR data information as the Y Filer Plus kit; (2) containing high-resolution Y-SNP information, not just under one of the major haplogroups; (3) data can be obtained from the attached table. A total of 3333 individuals were collected from five high-resolution Y-SNP-STR studies as shown in electronic supplementary material, table S1 [12–16]. To facilitate data analysis and statistics, we unified SNPs and STRs to a consistent level (electronic supplementary material, table S2).

### 2.2. Quality control (QC)

All these five studies were performed in a laboratory accredited by the China National Accreditation Service for Conformity Assessment (CNAS). The Y haplogroup was named according to Y-DNA Haplogroup Tree 2019–2020 (https://isogg.org/tree/index.html). We analysed Y-STRs strictly following the recommendations of the DNA Commission of the International Society of Forensic Genetics (ISFG) [17].

### 2.3. Data analysis

#### 2.3.1. Y-SNP data analysis

Haplogroup frequency of each population was calculated by direct counting. Haplogroup diversity was calculated as: n(1-Σpi^2^)/(n-1) (Note: pi was the frequency of the ith haplogroup, and n was the sample size). Principal component analysis (PCA) was conducted based on the haplogroup frequencies in a previous study [15]. A total of 91 worldwide populations were selected for PCA to study the relationship between Chinese populations and other countries.

#### 2.3.2. Y-STR data analysis

Allele and haplotype frequencies were direct counted by Office Excel. The allele of DYS389b was obtained as: DYS389b = DYS389II-DYS389I. DYS385 and DYF387S1 were treated as allelic combinations. The gene diversity (GD) and haplotype diversity were calculated as: *n*(1 − Σpi^2^)/(*n* − 1) (Note: pi was the frequency of the ith alle/haplotype, and n was the sample size). Discrimination capacity (DC) was calculated as: DC = the number of observed haplotypes/the number of total samples. Match probability (MP) was computed as: MP = Σpi^2^. The pairwise genetic distances of *F*_ST_ were calculated using Arlequin 3.5.2 with 23 Y-STRs (excluding double allele loci: DYS385 and DYF387S1).

Time to the most recent common ancestor (TMRCA) was estimated based on Y-STR using BATWING (http://www.maths.abdn.ac.uk/~ijw). In this study, minor modifications were applied to the genetic and population parameters according to a previous study [18]. Genealogical mutation rates for the Y-chromosome were more reliable for estimating historical lineages with BATWING [19]. In this study, mutation rates of 14 Slowly Mutating Y-STRs (DYS389I, DYS389b, DYS390, DYS391, DYS392, DYS393, DYS437, DYS438, DYS439, DYS448, DYS456, DYS458, DYS635, YGATAH4) were set as in a previous study [11,20]. Two million Markov chain Monte Carlo (MCMC) samples were collected per run, after the first 3000 samples were abandoned as ‘burn-ins’. The statistical analysis was conducted using R package 3.4.0. The generation time was set to 25 years [21].

#### 2.3.3. Y-SNP-STR

##### 2.3.3.1. The correlation of STR with haplogroup

To extensively explore the correlation of STR and haplogroup, corresponding GD and frequency values of single-copy Y-STRs with different haplogroups was analysed by R software.

##### 2.3.3.2. Network

To present median-joining networks of these five populations, 23 single-locus STRs (excluding DYS385 and DYF387S1) and 143 Y-SNPs were imported into Network 5.0 [22]. The high weights of Y-SNPs were assigned to 99. The weights of Y-STRs were assigned from 1–5 according to mutation rates in YHRD (https://yhrd.org/pages/resources/mutation_rates) [1]. The optional pre-processing method was used to simplify the Y-haplotype data, and the Median Joining (MJ) method was used to optimize the results calculated. Network Publisher was used to draw pie charts by editing its colours, line thicknesses and font styles.

## 3. Results

### 3.1. Y haplogroup distribution

**Figure 1.**
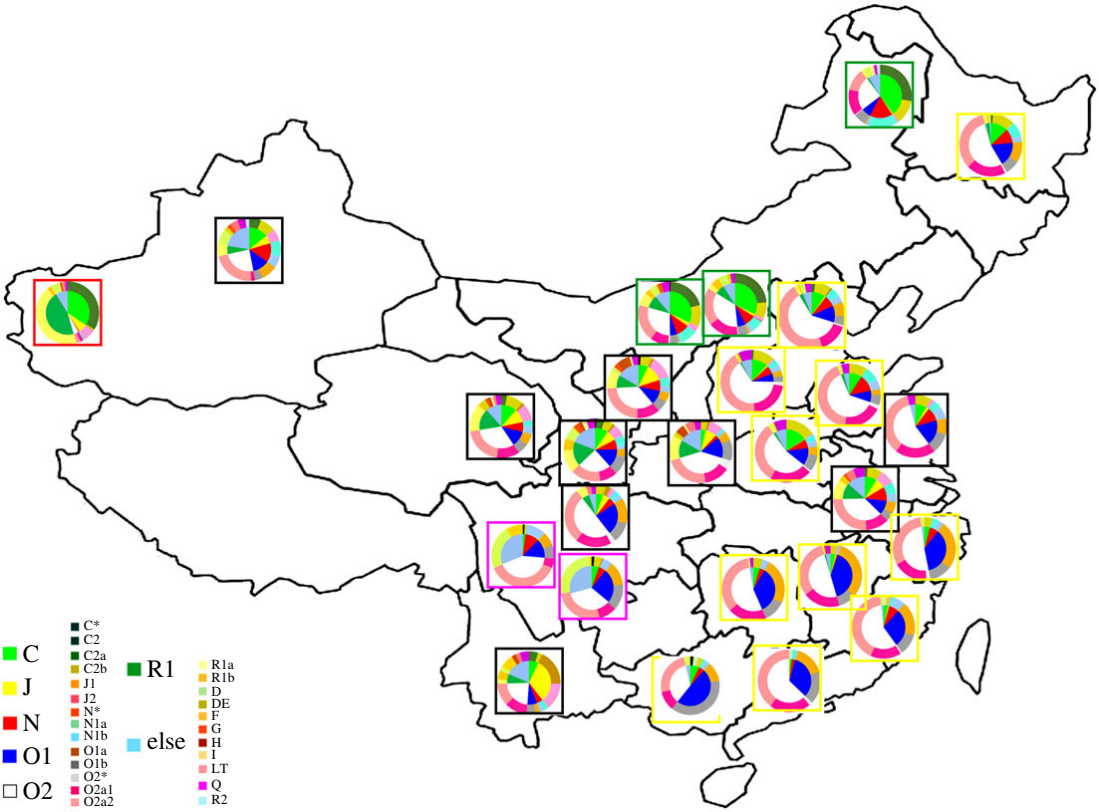
Geographical locations and haplogroup distribution of sampling including 11 Han (marked by Yellow Box), 9 Hui (marked by Black Box), 3 Mongolia (marked by Green Box), 2 Yi (marked by Purple Box) and 1 Kyrgyz (marked by Red Box) populations in China.

The highest haplogroup diversity was found in the Hui population (0.979), followed by Han (0.966), Mongolia (0.956), Yi (0.910) and Kyrgyz (0.791). As shown in table 1, five major Y-chromosomal haplogroups in China were O-M175, C-M130, R-M207, N-M231, and D-M174. The most predominant haplogroup of Han, Hui, Mongolia and Yi was haplogroup O-M175, but its frequency was different in these four populations (Han:0.800, Yi:0.583, Hui:0.474 and Mongolia:0.365), while the most predominant haplogroup of Kyrgyz was haplogroup R-M207 (0.455). The haplogroup frequencies in different geographical regions were shown in figure 1. It showed that the differences in haplogroup frequencies not only existed among ethnic groups, but also in geographical locations. The frequency of haplogroup O1 was significantly higher in the southeastern coastal regions than in the northern regions of China. The frequency of haplogroup C was significantly higher in the northern regions than in the southern regions.

**Table 1.** Frequency of haplogroup with five ethnic groups in China.

	C	DE	D	F	G	H	I	J	LT	N	O	P	Q	R
all	0.156	0.009	0.050	0.007	0.005	0.005	0.004	0.030	0.005	0.072	0.548	0.001	0.024	0.083
Han	0.083	0.013	0.002		0.001			0.004		0.062	0.800		0.026	0.010
Hui	0.083	0.012	0.026	0.005	0.015	0.023	0.011	0.097	0.020	0.075	0.474		0.040	0.118
Mongolia	0.346	0.009	0.041		0.007		0.006	0.018	0.001	0.115	0.365		0.027	0.065
Yi	0.037		0.244	0.049						0.077	0.583	0.009		
Kyrgyz	0.347		0.048		0.006	0.006	0.003	0.067	0.013	0.006	0.035		0.013	0.455

**Figure 2.**
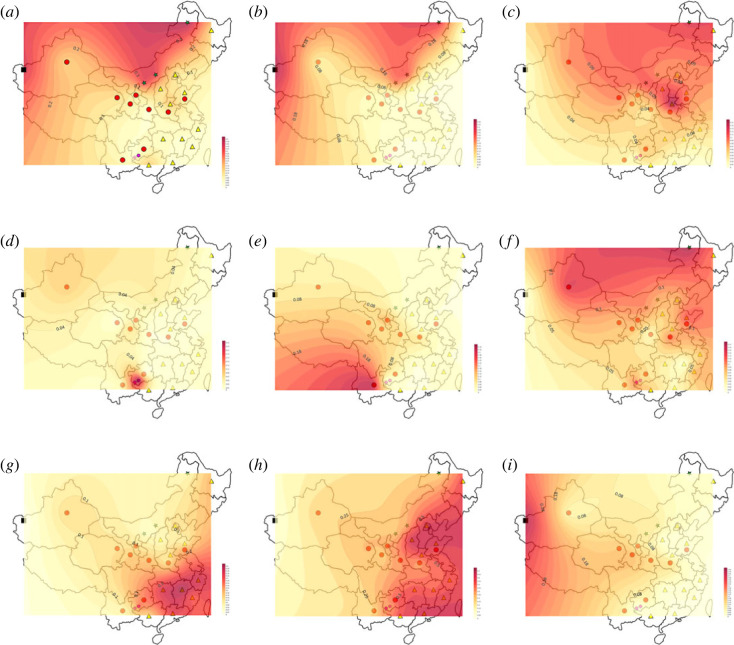
Contour map of haplogroup frequencies in Chinese. (*a*) The contour map of haplogroup frequencies of C. (*b*) The contour map of sub-haplogroup frequencies of C2a. (*c*) The contour map of sub-haplogroup frequencies of C2b. (*d*) The contour map of haplogroup frequencies of D. (*e*) The contour map of haplogroup frequencies of J. (*f*) The contour map of haplogroup frequencies of N. (*g*) The contour map of haplogroup frequencies of O1. (*h*) The contour map of haplogroup frequencies of O2. (*i*) The contour map of haplogroup frequencies of R.

In order to further explore the haplogroup distribution trend, the frequency contour plots with haplogroup were conducted. The results showed that haplogroup C mainly distributed in northern China, with decreasing haplogroup frequency to the south (figure 2*a*). Intensive analysis of haplogroup C showed that the frequency of sub-haplogroup C2a-L1373 decreased from north to south (figure 2*b*). However, a higher frequency of sub-haplogroup C2b-F1067 was found in Henan Han (figure 2*c*). Haplogroup D showed higher haplogroup frequency in the Yi population, followed by the northwestern Hui group, which may be related to the geographical proximity of Tibet (figure 2*d*). The frequency of haplogroup J showed a decreasing trend from southwestern China to northern China (figure 2*e*). A higher frequency of haplogroup N exists in northern China and decreases southwards along the coast (figure 2*f*). The highest frequency of haplogroup O1-F265 was found in the southeast Han (greater than 25%) and it decreases gradually toward the northwest (figure 2*g*). Haplogroup O2-M122 is widely distributed in the eastern coastal region of China, and its frequency gradually decreased from the east to the west (figure 2*h*). Haplogroup R-M207 possessed a high frequency in western China and gradually declined toward eastern China (figure 2*i*).

Further analysis of sub-haplogroups C and O revealed that different sub-haplogroups existed in these five ethnic groups. In haplogroup C, most of the Kyrgyz distributed in sub-haplogroup C2a-L1373, while most of the Han and Yi distributed in sub-haplogroup C2b-F1067 (electronic supplementary material, figure S1). In haplogroup O, the highest haplogroup diversity was Hui (0.956), followed by Han (0.951), Mongolia (0.925), Yi (0.887) and Kyrgyz (0.793). Interestingly, no haplogroup O1-F265 was found in Kyrgyz (electronic supplementary material, figure S2). We found that the frequency of haplogroup O1a-M119 decreased from southeastern to northwestern China, while haplogroup O1b spread from southern to northern China according to the results of haplogroup frequency contour analysis (electronic supplementary material, figure S3*a*, electronic supplementary material figure, S3*b*). The frequency of haplogroup O2a1 showed a decline from eastern to western China (electronic supplementary material, figure S3*c*). The frequency of haplogroup O2a2 showed a decreasing trend from eastern to northwestern China (electronic supplementary material, figure S3*d*).

### 3.2. Haplotype diversity and genetic diversity of Y-STRs

Haplotypes containing 27 Y-STRs from these five ethnic groups were shown in electronic supplementary material, table S2. In this study, a total of 3139 haplotypes were found. No shared haplotype existed in different ethnic groups. The Y-STR haplotype frequencies of Han, Hui, Mongolia, Yi, and Kyrgyz ranged from 0.00079 to 0.00158, from 0.00153 to 0.00462, from 0.00147 to 0.01178, from 0.00234 to 0.00703, and from 0.00318 to 0.01911 respectively. Higher haplotype diversity was found in the Han population (0.99999), followed by Hui (0.99987), Yi (0.99977), Mongolia (0.99950) and Kyrgyz (0.99803). The highest DC was found in the Han population (0.998), followed by Hui (0.960), Yi (0.953), Mongolia (0.878), and Kyrgyz (0.793). The MPs of these five ethnic populations were 0.0008, 0.0017, 0.0020, 0.0026 and 0.0052, respectively.

Gene diversities for the 27 Y-STR loci were shown in electronic supplementary material, table S3 and electronic supplementary material, table S4. DYS385 was the highest polymorphic marker overall (GD = 0.967), while DYS391 showed the least diversity (GD = 0.435) overall. Interestingly, the GD values of DYS385 ranked first in Han, Hui, and Yi, while DYF387S1 ranked first in Kyrgyz and Mongolia. The GD values of DYS391 ranked bottom in Han and Hui, while DYS437 ranked bottom in Kyrgyz, Mongolia and Yi.

### 3.3. Y-SNP-STR analysis

#### 3.3.1. The correlation of STR with haplogroup

Based on the GD of Y-STR, we found that DYS389b, DYS460, DYS390, DYS481, DYS533, DYS389I and DYS449 were significantly associated with haplogroup F (electronic supplementary material, figure S4), while DYS439 and DYS518 were significantly associated with haplogroup H. In an attempt to further explore the correlation of STRs within each haplogroup, alleles of STRs were analysed by different haplogroups (electronic supplementary material figure, S5). Using the frequency of alleles, we achieved a significant correlation between Y-STRs and haplogroups (electronic supplementary material, table S5). The above results suggested that the specific Y-STR haplotype can be used to predict haplogroups, which can improve the precision of existing Y haplogroup prediction software, especially in East Asian populations.

#### 3.3.2. Network

**Figure 3.**
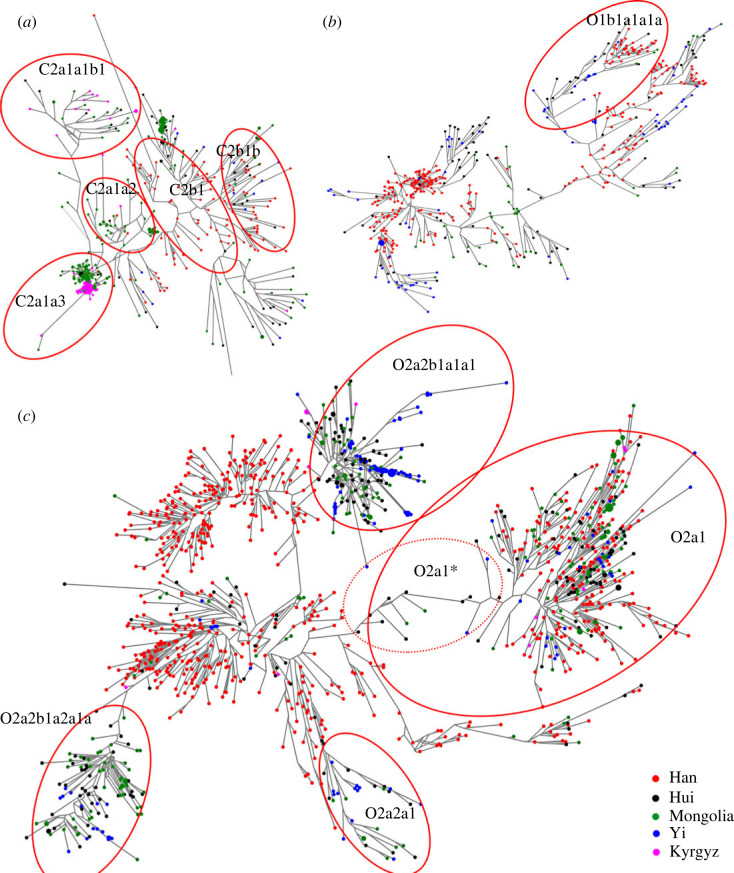
Network for these five ethnic groups using 23 Y-STR and 143 Y-SNP. The size of node was according haplotype frequency. (*a*) The network of haplogroup C-M130. (*b*) The network of haplogroup O1-F265. (*c*) The network of haplogroup O2-M122.

We applied Network analysis to further infer the possible patriarchal activities within China. Mongolia, Kyrgyz and some Northwest Hui were clustered in C2a1a1b1-F1756, C2a1a2-M48 and C2a1a3-M504, while Han populations distributed in C2b1-Z1338 and C2b1b-F845 (figure 3*a*). Interestingly, we found that haplogroup C* only distributed in southern China. Further analysis of sub-haplogroup C2a1a3-M504, the result showed that the Mongolian and Kyrgyz individuals divided into two clusters, indicating that the sub-haplogroup C2a1a3-M504 in the Kyrgyz was different from in Mongolians. Star-like networks of haplogroup D-M174 in electronic supplementary material, figure S6 showed that the sub-haplogroup D presented in the Han, Hui, Mongolia and Kyrgyz populations might derive from Yi-related ancestral populations. Further analysis of sub-haplogroup D showed that the Yi population expanded in haplogroup D1a1a1a1b-SK541, while the Kyrgyz population only distributed in D1a1b1a-M533. No haplogroup J-M304 was found in the Yi population (electronic supplementary material, figure S7). High polymorphism of haplogroup J was found in the Hui population. High polymorphism of haplogroup N-M231 was found in the Han population (electronic supplementary material, figure S8). Most Yi individuals clustered in haplogroup-N1b, while Mongolians distributed in N1a. In sub-haplogroup N1a1a1a1a3-B197, Mongolians was clustered and expansion. However, in haplogroup N1a2-F1008, Han individuals and Mongolians distributed in different branches which indicated that the two populations have different origins. No Kyrgyz individual was found in haplogroup O1-F265 (figure 3*b*). Yi, Hui and Mongolia populations distributed in the downstream of haplogroup O1- F265, except sub-haplogroup O1b1a1a1a-F1252. In sub-haplogroup O2a1-L467, these five populations distributed alternately. But in the O2a1*, only Hui and Mongolian individuals appeared (figure 3*c*). In haplogroup O2a2-P201, most Hui, Mongolian, Yi and Kyrgyz individuals distributed the downstream of the central reference Han people, such as sub-haplogroup O2a2a1-F2588, O2a2b1a1a1-F438 and O2a2b1a2a1a-F46. It suggested that haplogroups O2a1-L467 and O2a2-P201 have different origins in Chinese populations. Only Hui and Mongolians distributed in R2-M479 (electronic supplementary material, figure S9). Star-like networks of haplogroup R1a1a1b2a2-Z2124 were observed in Kyrgyz individuals, which reflected likely population expansion happening in these people. No Yi population was found in haplogroup R-M207. In the Chinese Han population, haplogroup R was mainly distributed in the northern Han.

#### 3.3.3. Time estimation for major haplogroups

The TMRCA for the Chinese populations can be traced to 17 kya (electronic supplementary material, table S6). The TMRCA of haplogroup C with Mongolia, Kyrgyz and Northwest Hui (approx. 13.6–17.5 kya) predated other groups. Further analysis of sub-haplogroups C2a and C2b showed TMRCA can be dated back to 7.6 and 14.5 kya in the Mongolian group. TMRCA of haplogroup N in the northern Chinese populations was earlier than that of the southern populations, which was similar to the results of the frequency distribution contour heat map. It indicated that haplogroup N is spreading from north to south. Although higher frequency of haplogroup O1a was found in southern China, the TMRCA of Northwest Hui groups can trace to 12.2–16.7 kya.

#### 3.3.4. The structure within Chinese populations

To reveal the substructure within Chinese populations, pairwise F_ST_ values were calculated by using 23 Y-STRs (excluding DYS385 and DYF387S1, electronic supplementary material, figure S10). The results showed that Kyrgyz was the most distantly related to other populations (*p* < 0.05). The Hui populations were divided into three groups: Cluster 1 (Gansu Hui, Qinghai Hui, Xinjiang Hui, Ningxia Hui, Henan Hui and Shannxi Hui), Cluster 2 (Sichuan Hui and Shandong Hui) and Yunnan Hui. The small genetic distance was shown in Cluster 1 (F_ST_ < 0.0027). Cluster 2 Hui populations were closer to Northern Han populations than other Hui populations. Yunnan Hui were closer to Yi populations than other Hui populations. Chinese Han populations were separated into two clusters: Northern Han and Southern Han.

In a principal component analysis (PCA) of these five ethnic groups, similar genetic profiles were observed as F_ST_ (electronic supplementary material, figure S11). Han, Kyrgyz and Hui were separated with PC1. Northern Han and Southern Han were separated with PC2. From the quadrant perspective, Cluster 1 Hui populations were clustered with Mongolians, while Cluster 2 Hui populations were clustered with Chinese Han.

#### 3.3.5. The genetic relationships with populations of other countries

**Figure 4.**
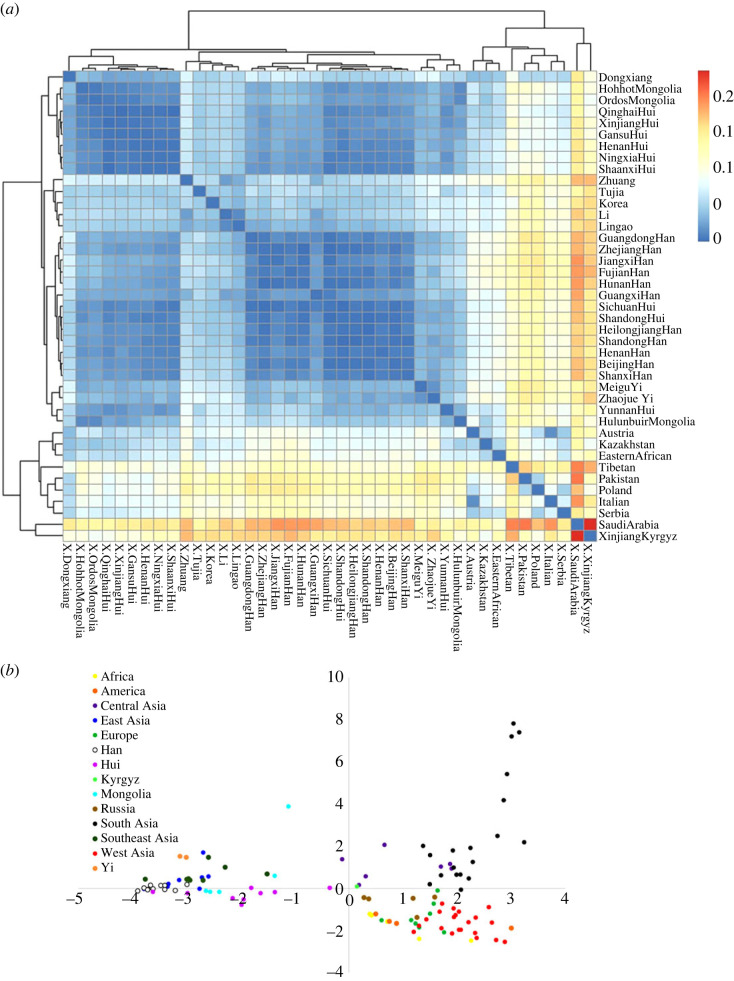
Relationship between populations in this study and other worldwide references. (*a*) Heatmap of genetic distance between populations in this and ancient references. (*b*) PCA result showed an overview population relationship between populations in this and ancient references.

To better understand the Chinese genetic flow and mixture, the relationships of populations in this study and 15 reference populations were explored via 23 single locus Y-STR (figure 4*a*). We found that Cluster 1 Hui populations had closer genetic distance with Mongolia and Dongxiang populations. Han populations were clustered with the other Hui groups, Yi and Lingao populations. To further illustrate the genetic relatedness of the studied populations and worldwide populations, PCA analyses were conducted by 34 Y-SNP markers and 91 populations (figure 4*b*). The results showed that Han, Hui, Mongolia, Yi, Southeast Asian and other East Asians clustered in the left quadrant. South Asians and central Asians were located in the upper right quadrant. West Asians, Europeans and Africans were located in lower right quadrant. However, Kyrgyz located at the centre, which indicated that Kyrgyz population exhibited a genetic admixture of Asian and European populations.

## 4. Discussion

Historically, China has experienced a number of population migrations, such as the migration of nomads from the north to the interior during the late Eastern Han Dynasty, and the mass migration of northerners to the south to escape the war during the An-Shi Rebellion. Therefore, population genetic structure and population migration have been a hot topic in Chinese genetic research. In this study, we explored the genetic affinities of the Han, Hui, Mongolia, Yi, and Kyrgyz populations to each other and to neighbouring populations using a variety of analytic methods, and investigated the values of Y chromosome markers (both Y-SNP and Y-STR) in biogeographical ancestry inference.

The population comparisons by PCA showed three intercontinental clusters: Cluster 1: East and Southeast Asian populations; Cluster 2: South and Central Asian populations; Cluster 3: African, American and European populations. In this study, we found that Han and Yi populations were clustered with East and Southeast Asian populations. It was consistent with Cheng *et al*.'s study [23]. Hui and Mongolia populations presented a multi-ethnic mixed character. Kyrgyz was clustered with Central Asian populations. According to historical records, many Muslims from Persia, Arabia and Central Asia entered China via the Silk Road during the Tang Dynasty. The governments of the Tang, Song and Yuan Dynasties encouraged immigration to settle and establish businesses in the localities [24].

Haplogroup O-M175 is the most common paternal lineage in East Asian and Southeast Asian populations [25]. It was also the dominant haplogroup in our study, except for the Kyrgyz population. Haplogroup O1a-M119 is dominant in Austronesian-speaking populations of Southeast Asian islands, but rare in Austronesian-speaking populations from the Pacific islands [26]. Previous studies have showed that haplogroup O1a is one of the paternal lineages of the Han, Tai-Kadai-speaking and Austronesian-speaking populations. It spread to Southwest China and Taiwan between 4.5 and 6 kya [27]. In this study, a higher frequency of haplogroup O1 was found in South China, which was consistent with Ding *et al*. study [28]. Interregional migration accompanied by high genetic drift rates and genealogical loss in northern populations could explain the asymmetry in genealogical composition, which indicates that northern origins cannot be ruled out. In our study, South China populations in haplogroup O1a showed relatively young ages, suggesting ancient migrations from North China into South China via the northern route.

Ding *et al*. found that O2-M122 haplotypes in southern East Asia were more diverse than that in northern East Asia by typing 2,332 East Asian individuals, suggesting that haplogroup O2 originated in the south and migrated northward [29]. The frequency of haplogroup O2 among the 26 populations ranged from 0.038 to 0.657, showing a decreasing trend from eastern to western China. O2a1-L467 and O2a2-IMS-JST021354 are the two major subseries of haplogroup O2, which have similar expansion time around 10 and 12.2kya, respectively. Rice, broomcorn millet and foxtail millet had been planted roughly 10kya in China, which may accelerate the expansion of the Chinese people.

Haplogroup C-M130 is the strong supporting evidence for the migration and settlement of modern humans from the Middle East to South and East Asia [30]. They proposed that Southeast Asia may be the cradle of the C-M130 lineage and experienced population expansion. Some studies tended to support the view that individuals derived from C2-M217 first reached South Asia and then migrated via two routes: Central Asia and Southeast Asia [31]. In this study, haplogroup C-M130 and C2-M217 concentrated in southern China (figure 2), which supported the South Asian theory. C2a-F1396 and C2b-F1067 are the main branches of the C haplogroup in China, especially in northern China. In 2020, Wu pointed that C2a-F1396 was from Northern Asia, while C2b-F1067 was from East Asia [32]. But in our study, higher frequency and diversity was found in Mongolians. In this study, haplogroups C can be traced back to 17 kya and show a north to south expansion around 10 kya.

A previous study showed that haplogroup D mainly distributed in East Asia, especially in Tibetan and Japanese (30%–40%) [31]. In this study, a higher frequency (greater than 5%) of haplogroup D was found in Yi, Gansu Hui and Xinjiang Hui, which may be due to geographical proximity.

Haplogroup R occurs at high frequency in modern Europeans, especially in Western Europe [33]. Although R1a1-M17 occurs across Eurasia, higher frequency was found in West Asia and Central-South Asia [34]. In this study, higher frequency (greater than 10%) was found in Northwest Hui (Qinghai, Ningxia and Gansu) and Kyrgyz, especially in Kyrgyz (45.5%). No haplogroup R was found in the South Han groups. The TMRCA of haplogroup R1a can trace back to around 15kya. The expansion time of the ancestry of haplogroup R1a was consistent with the fact that rice, broomcorn millet and foxtail millet had been planted in China, which may accelerate the expansion of haplogroup R [35,36].

The Y haplogroup is a valuable tool for studying the origin of humans and inferring the admixture among populations [37,38]. Due to the high cost and complex operation of next-generation sequencing, it has not commonly been used in grassroots forensic laboratories. The single base extension technique has demonstrated the ability to multiplex in the range 6–34 SNPs in a single PCR reaction with capillary electrophoresis platform [39]. However, it is mostly used in scientific research not practice, due to the tedious operation and poor stability. With the development of commercial Y-STR kits, Y-STR tests are widely used in forensic sexual assault cases and family tracing [40]. So, a number of haplogroup prediction tools with Y-STR exist, such as YPredictor, NevGen and Whit Athey's Haplogroup Predictor [41]. But the exact methodology is sometimes unclear. Most of these methods are based on genetic genealogical changes in Europe and America, but lack data of Y haplogroups and STR in Asia. Previous studies are comparatively small datasets that may give unreliable results. In order to address this problem, we included 3333 unrelated males from five studies with independent Y-SNP and Y-STR data to explore the prediction of haplogroups by Y-STR.

## 5. Conclusion

Human migration has always been a hot topic in genetics research. In this study, 3333 Chinese individuals (Han, Hui, Mongolia, Yi, and Kyrgyz) were analysed by Y-STR and Y haplogroup. We found that differences of Y haplogroup and Y-STR not only existed among ethnic groups, but also in geographical locations. Furthermore, combining analysis of haplogroup frequencies, geographical positions and TMRCA, it was shown that Chinese populations have undergone a complex process of population migration. The population migration was concurrent with global temperature increases after the Last Glacial Maximum.

## Data Availability

The datasets supporting this article have been uploaded as part of the electronic supplementary material [42].
